# Preparation, allergenicity analysis and nutritional evaluation of partially and extensively hydrolyzed whey protein peptides

**DOI:** 10.1038/s41538-026-00778-8

**Published:** 2026-03-10

**Authors:** Shulin Zheng, Renyi Zhang, Xiao Chen, Meng Du, Hongyan Li, Jing Wang, Baoguo Sun

**Affiliations:** Key Laboratory of Geriatric Nutrition and Health, Beijing Technology and Business University, Ministry of Education, Beijing, China

**Keywords:** Biochemistry, Biotechnology

## Abstract

With the growing focus on health, investigating the potential benefits of whey protein peptides for musculoskeletal health is essential. This study employed enzymatic hydrolysis to prepare concentrated whey protein peptides. The results indicated that optimal conditions for partially hydrolyzed peptides were achieved using alcalase (1500 U/g for 0.5 h), while extensive hydrolysis was produced using alcalase and flavourzyme (3000 U/g, 1:1 ratio for 2.0 h). Post-hydrolysis, there was no amino acid loss, and branched-chain amino acid content increased by 56.68 mg/g; allergenicity of α-LA and β-LG decreased by 90.73% and 70.48%, respectively; treatment of Caco-2 cells with hydrolyzed peptides at 0.5-2.5 mg/mL for 24 h showed cell viability over 100% relative to controls, indicating non-cytotoxicity; the highest calcium transport efficiencies were 5.38 μg/well and 8.62 μg/well at 2.5 mg/mL. This study provides theoretical and technical support for hypoallergenic functional foods targeting musculoskeletal health.

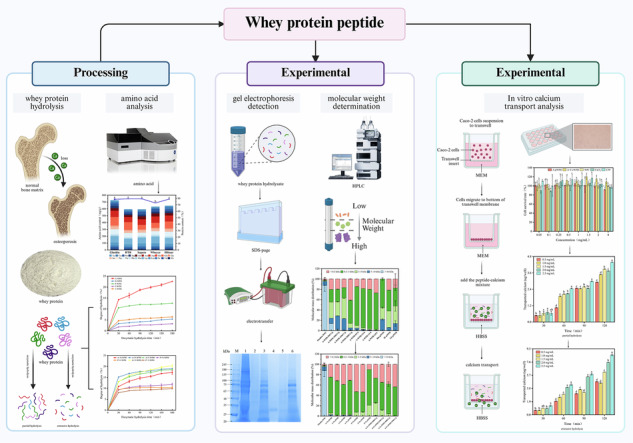

## Introduction

With the acceleration of global aging, musculoskeletal health has become an urgent global public health issue^1^. This multifaceted problem primarily involves two age-related diseases: osteoporosis and sarcopenia. Although they share overlapping risk factors (e.g., aging, sedentary behavior, and nutritional deficiencies), there are fundamental differences in their pathological mechanisms. Osteoporosis is characterized by reduced bone mineral density (BMD) and deteriorated bone microstructure, driven by an imbalance between osteoclastic bone resorption and osteoblastic bone formation^2^. Key risk factors include long-term calcium/vitamin D insufficiency, postmenopausal estrogen decline, chronic low-grade inflammation (mediated by cytokines such as TNF-α and IL-6), and impaired bone turnover regulation^3,4^. In contrast, sarcopenia refers to the progressive loss of muscle mass, strength, and functional capacity, mainly attributed to age-related decreases in muscle protein synthesis (associated with reduced mTOR pathway activation), increased muscle protein breakdown (mediated via the ubiquitin-proteasome system), and diminished satellite cell proliferation and differentiation capacity^5,6^. Both conditions severely impair quality of life and increase the risk of fractures, falls, disability, and mortality in older adults^7^.

In addition to pharmacological interventions (e.g., bisphosphonates for osteoporosis and resistance training for sarcopenia), lifestyle modifications especially dietary management play a crucial role in the prevention and treatment of musculoskeletal diseases^8,9^. Dietary supplements targeting musculoskeletal health have received widespread attention, with their core lying in the promotion of bone and muscle health by synergistically acting bioactive ingredients^10^. Among these, calcium and vitamin D remain the foundation for maintaining bone mineralization and muscle contractile function, while bioactive peptides (e.g., casein phosphopeptides, CPP) have emerged as highly promising candidates due to their ability to improve calcium solubility, inhibit calcium precipitation, and enhance intestinal calcium absorption^11,12^. Whey protein-derived peptides exert dual structural and metabolic support: they not only provide essential amino acids (e.g., leucine) to stimulate muscle protein synthesis but also act as calcium carriers to improve calcium bioavailability^13^. Dairy products are the main dietary sources of calcium and high-quality protein; however, their intake is limited in specific populations due to milk protein allergy (MPA) and lactose intolerance^14^. MPA is an immunoglobulin E (IgE)-mediated hypersensitivity reaction triggered by multiple whey protein allergens, among which α-lactalbumin (α-LA) and β-lactoglobulin (β-LG) are the major pathogenic factors (accounting for approximately 80% of clinical reactions in MPA patients)^15,16^. In addition, minor allergens such as lactoferrin, bovine serum albumin (BSA), and immunoglobulin G may also elicit allergic responses in highly sensitive individuals, though their clinical relevance is relatively low^17^. Individuals with lactose intolerance, lacking sufficient lactase activity to hydrolyze lactose, often avoid consuming dairy products, which may lead to calcium and protein deficiencies^18^. Therefore, the development of hypoallergenic functional ingredients capable of enhancing calcium absorption is crucial for meeting the nutritional needs of these underserved populations^19^.

As a by-product of cheese production, whey protein is recognized as a “high-quality protein” due to its complete amino acid composition, high digestibility, and abundant content of bioactive peptides^20^. However, its inherent allergenicity (primarily derived from α-LA and β-LG) and large molecular weight limit its application in functional foods for sensitive populations^21^. Enzymatic hydrolysis is a well-established technique for modifying the properties of whey protein: it reduces allergenic potential by cleaving linear/conformational allergenic epitopes, generates small-molecular-weight peptides with enhanced bioactivity, and improves solubility and digestibility^19^. Previous studies have shown that whey protein hydrolysates possess various biological activities, including antihypertensive, antioxidant, and calcium-binding effects^21^. For example, the dipeptide Phe-Asp (FD) from whey protein hydrolysates has been proven to bind tightly to calcium via the carboxyl groups of aspartic acid residues and inhibit calcium phosphate precipitation, thereby promoting calcium absorption^22^. Despite these advances, critical knowledge gaps remain in the field. Most studies focus solely on partial or extensive hydrolysis processes, lacking systematic comparisons of how hydrolysis degree (partial and extensive) affects peptide size distribution, allergenicity, and calcium transport efficiency. It remains unclear how to balance nutritional retention, allergenicity reduction, and bioactivity enhancement through optimal hydrolysis degree. Alcalase is a serine endopeptidase that can cleave internal peptide bonds (preferentially acting on hydrophobic amino acids) to generate medium-sized peptides (3–10 kDa); while flavourzyme is a mixture of endopeptidases and exopeptidases that can further cleave N-terminal and C-terminal amino acids to produce small peptides (<3 kDa); the synergistic effect of the two on peptide composition and function has not been fully elucidated. The quantitative relationship between peptide size and calcium transport activity is still unclear.

This study aims to systematically investigate the effects of partial and extensive hydrolysis on the nutritional quality, allergenic potential, and calcium transport capacity of whey protein peptides. Compared with partial hydrolysis using alcalase alone, extensive hydrolysis (alcalase and flavourzyme) will increase the proportion of small peptides (<3 kDa), thereby enhancing calcium transport across Caco-2 monolayers by improving calcium solubility and interacting with intestinal transporters. Enzymatic hydrolysis will significantly reduce the allergenic potential of α-LA and β-LG, and extensive hydrolysis will achieve a greater reduction effect than partial hydrolysis due to more complete cleavage of epitopes. Hydrolyzed whey protein peptides will retain the essential amino acid composition (no statistically significant loss *p* > 0.05), maintaining high nutritional value. This study provides a theoretical basis and technical support for the development of hypoallergenic, high-nutrition calcium absorption enhancers for functional foods targeting musculoskeletal health.

## Results and discussion

### Selection of whey protein raw materials

**Fig. 1:**
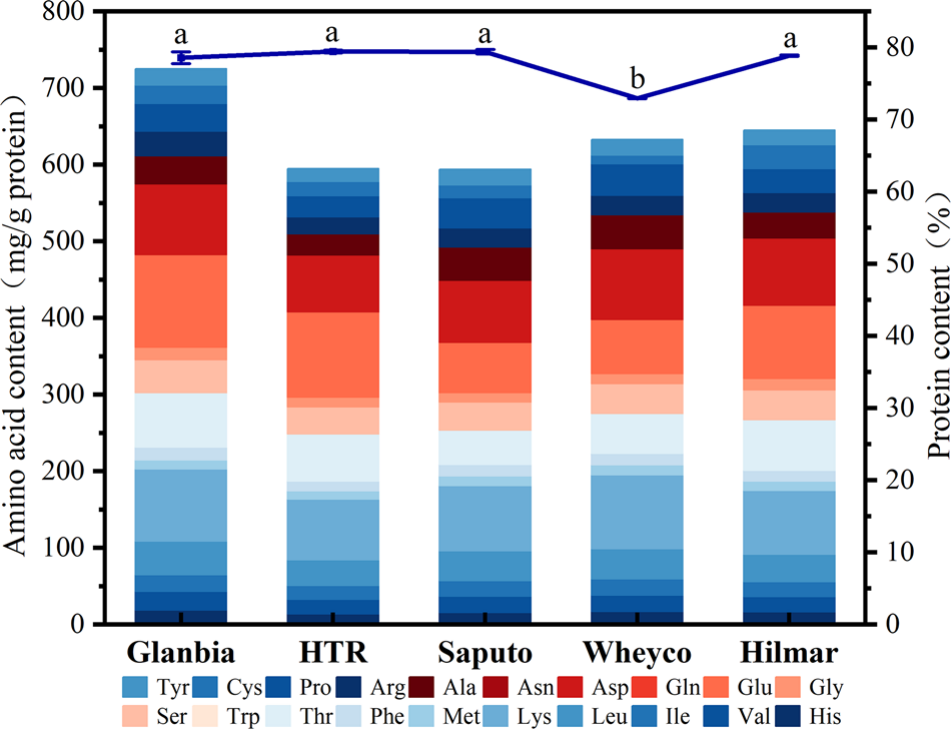
Analysis of amino acid composition distribution and protein content in five types of WPC. Data are expressed as mean ± standard deviation (SD, *n* = 3). Different lowercase letters (**a**, **b**) indicate significant differences among groups (*p* < 0.05, one-way ANOVA with Tukey’s post-hoc test).

Commercially available whey protein concentrate (WPC) products vary widely, and five types of WPC with high popularity, recognition, and usage (Glanbia, Fonterra HTR, Saputo, Wheyco, and Hilmar) were selected for raw material screening. Tables S1, S2, and S3 present comparisons of protein proximity and essential amino acid scores (RAA, RC, SRC, and EAAI) for five types of WPC and two hydrolyzed peptides, respectively. Results of amino acid analysis and protein content determination are shown in Fig. 1 and Table S2. According to the amino acid analysis, all WPC samples were rich in 17 amino acids. Glanbia exhibited the highest protein content, total amino acid (TAA) content (725.04 mg/g), essential amino acid (EAA) content (302.42 mg/g), and branched-chain amino acid content (89.64 mg/g). The ratios of EAA to TAA (0.42) and EAA to non-essential amino acids (NEAA, 0.72) were closer to the FAO/WHO proposed standards (EAA/TAA: 0.400 and EAA/NEAA: 0.600).

As shown in Table 1, among the five types of WPC, only protein content exhibited differences, while other basic parameters showed no significant differences. According to the fuzzy recognition method^23^, Table S1 presents the protein similarity values of five types of WPC. From the results in Table S1, it can be observed that μ1 > μ3 > μ5 > μ4 > μ2. Glanbia protein exhibits the highest closeness degree of 0.801, which is closer to the standard value of 1 for egg protein. This also demonstrates that it outperforms other WPCs in terms of nutritional value. The RC method, designed based on the amino acid balance theory, is a method for evaluating protein nutritional value that includes two patterns: the Egg Pattern and the FAO/WHO Pattern. Using the formula, the evaluation results of EAAI, RAA, RC, and SRC for the five types of WPC were calculated, as shown in Table S2 and Table S3. It is generally believed that the closer the values are to the two standard patterns, the higher the nutritional value of the WPC that can be utilized by the human body. For the whole egg pattern, Relative RAA and RC values close to 1 indicate good matching of EAA composition; RC > 1 denotes EAA excess, while RC < 1 indicates EAA deficiency. SRC value approaching 100 reflects high nutritional value and favorable digestibility. Based on the EAAI, proteins are categorized as high-quality (EAAI > 0.95), good-quality (0.85 < EAAI ≤ 0.95), usable (0.75 ≤ EAAI ≤ 0.85), or unusable (EAAI < 0.75).

**Table 1:** Basic parameters of five commercially available Whey Protein Concentrate (WPC) brands

	Moisture content	Protein %(Kjeldahl)	Solubility variation
Glanbia	4.55 ± 0.13^a^	78.56 ± 0.81^a^	98.56 ± 0.54^a^
HTR	4.23 ± 0.11^a^	79.43 ± 0.18^a^	96.93 ± 0.36^a^
Saputo	3.87 ± 0.08^a^	79.40 ± 0.32^a^	97.31 ± 0.23^a^
Wheyco	4.15 ± 0.15^a^	72.96 ± 0.05^b^	95.48 ± 0.41^a^
Hilmar	3.96 ± 0.35^a^	78.86 ± 0.09^a^	96.68 ± 0.18^a^

Different lowercase letters (a, b) indicate significant differences among groups (*p*<0.05)

According to the amino acid composition analysis of the egg evaluation pattern in Table S2, the SRC and EAAI results of the nutritional evaluation of these five types of WPC are relatively low. Among them, Glanbia has an EAAI of 0.63 and an SRC of 39.69, which is the highest under this pattern, and the RC and RAA of other essential amino acids are partially distributed. Fonterra (HTR) has an EAAI of 0.51 and an SRC of 35.66, with overall lower indicators. Wheyco has an EAAI of 0.54 and an SRC of 47.47, but the RAA values of leucine (Leu) and isoleucine (Ile) are relatively low. The EAAI values of Hilmar and Saputo are 0.55 and 0.57, respectively, and their SRC values are both lower than that of Glanbia, and the balance of valine (Val) and phenylalanine + tyrosine (Phe+Tyr) amino acids is slightly inferior. Wheyco’s SRC of 47.47 represents the optimal proportion, and Glanbia’s EAAI of 0.63 represents the best balance, among which Glanbia has a better relative balance of RC and RAA for each amino acid.

According to the amino acid composition evaluation of the FAO/WHO pattern in Table S3, Wheyco’s SRC of 53.09 and Glanbia’s EAAI of 0.85 also have advantages. Glanbia leads in EAAI under both patterns, indicating that the geometric mean of its essential amino acids is closer to the ideal state, making it suitable for long-term nutritional support.

### Optimization of hydrolysis conditions for whey proteins

**Fig. 2:**
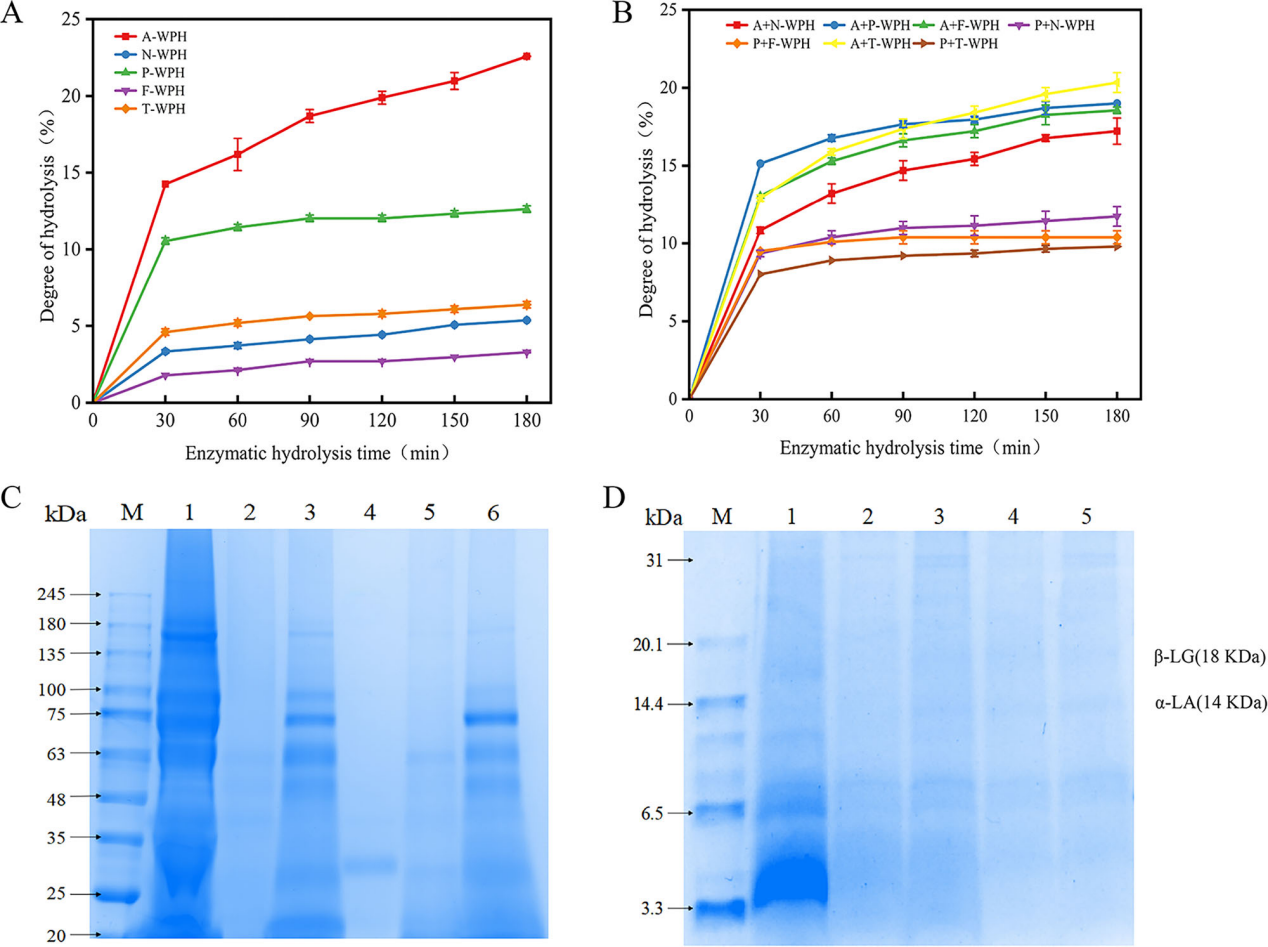
Degree of hydrolysis and protein profile of WPC hydrolyzed by different proteases and their combinations. **A** Degree of hydrolysis of WPC hydrolyzed by different proteases. **B** Degree of hydrolysis of WPC hydrolyzed by different combinations of proteases. **C** SDS-PAGE of products from the hydrolysis of WPC by different proteases. Lane M is the protein marker. Lane 1 is the unhydrolyzed Glanbia (WPC). Lanes 2 to 6 are the hydrolyzed products by alcalase, neutrase, papain, flavourzyme and trypsin, respectively. **D** Tricine SDS-PAGE of WPC products hydrolyzed by different proteases. Lane M is the protein marker. Lanes 1 to 5 represent the hydrolysis products of alcalase, neutrase, papain, flavourzyme and trypsin, respectively.

Figure 2A illustrates the dynamic changes for the degree of hydrolysis (DH) over the enzymatic hydrolysis time during the hydrolysis of whey protein concentrate by single enzymes. WPHs hydrolyzed by different single enzymes, namely alcalase (A-WPH), neutrase (N-WPH), papain (P-WPH), flavourzyme (F-WPH), and trypsin (T-WPH), exhibited significant differences in hydrolysis efficiency. Among them, the DH of A-WPH increased remarkably during the enzymatic hydrolysis process and reached a relatively high level at 180 min, indicating strong hydrolytic activity. This might be related to its high affinity for specific peptide bonds in whey protein concentrate. The substrate specificity of proteases significantly affects their hydrolysis efficiency, and the preferential action of specific proteases on certain peptide bonds leads to differences in the hydrolysis rate^24^. In contrast, the increases in the degrees of hydrolysis of N-WPH, P-WPH, F-WPH, and T-WPH were relatively gentle. However, electrophoresis analysis in Fig. 2C and D indicates that A-WPH, P-WPH, and F-WPH exhibit superior potential in producing low-molecular-weight peptides through protease hydrolysis. Therefore, the above results will be comprehensively considered during the screening process of the combined enzymes.

Figure 2B represents the evolution trend of the degree of hydrolysis over the enzymatic hydrolysis time during the hydrolysis of whey protein concentrate by combined enzymes. Compared with single-enzyme hydrolysis, the DH of WPHs with various combined enzyme combinations, namely alcalase and papain (A + P-WPH), alcalase and trypsin (A + T-WPH), and alcalase and flavourzyme (A + F-WPH), was generally higher. Some combined enzyme combinations could reach higher degrees of hydrolysis within the same time, which strongly demonstrates the positive effect of the synergistic action between enzymes on improving the hydrolysis effect of whey protein concentrate. A combined enzyme system can act on different sites of proteins through different enzymes, overcoming the limitations of single-enzyme hydrolysis, thus decomposing proteins more effectively and enhancing the degree of hydrolysis^25^. Further validation of the advantages of composite enzyme technology in improving hydrolysis efficiency in the field of protein processing.

### Determination of partial and extensive hydrolysis

**Fig. 3:**
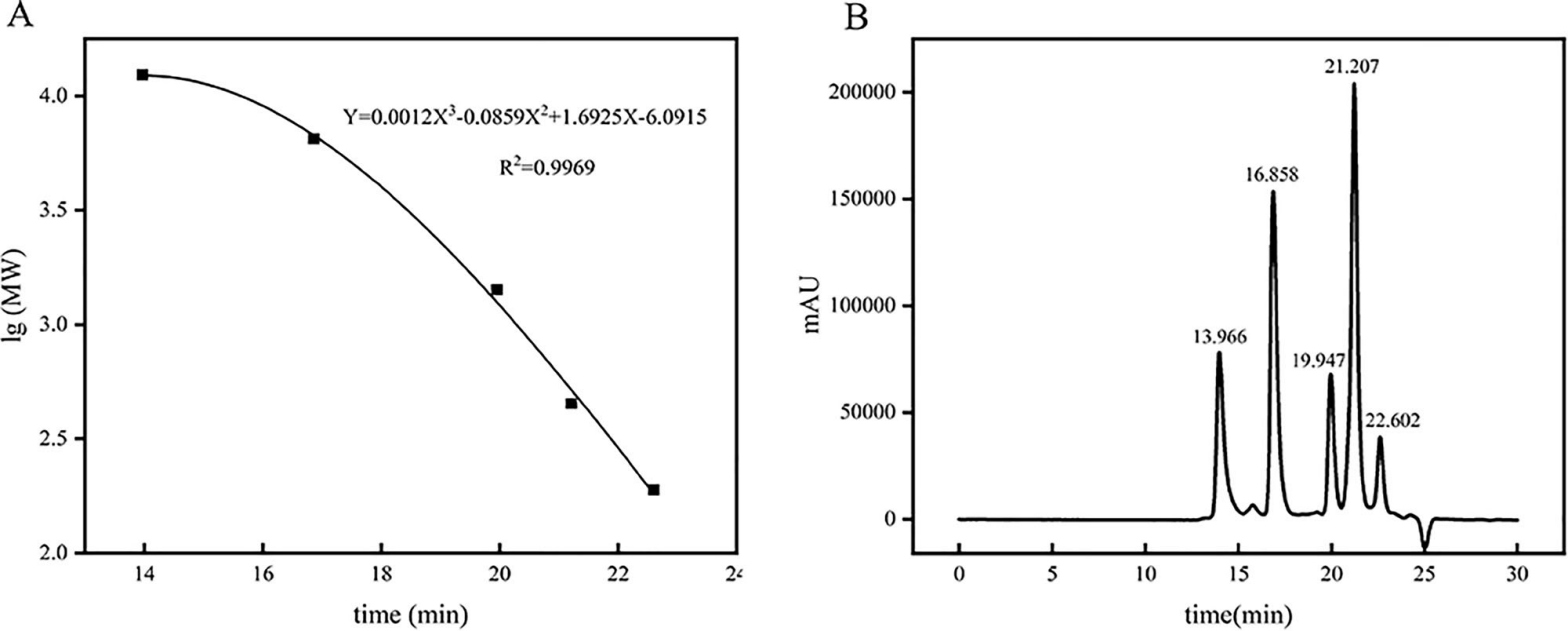
Molecular weight calibration and peak time profile of standard protein samples for size exclusion chromatography analysis. **A** Retention time and molecular weight logarithm calibration curve; **B** Peak time profile of standard samples.

As shown in Fig. 3B. the retention times and molecular weights of five standards (cytochrome C, peptidase, bacitracin, glycine-glycine-tyrosine-arginine, and glycine-glycine-glycine) are presented. Plotting the retention times of the standards on the x-axis and the logarithm of their molecular weights on the y-axis yields the standard curve. As shown in Fig. 3A. The standard curve for molecular weight calculation is: *y* = 0.0012*x*^3^− 0.0859*x*^2^ + 1.6925*x* − 6.0915, *R*^2^ = 0.9969. This standard curve exhibits excellent linearity, enabling the determination of the corresponding molecular weight value for any retention time based on the standard curve.

**Fig. 4:**
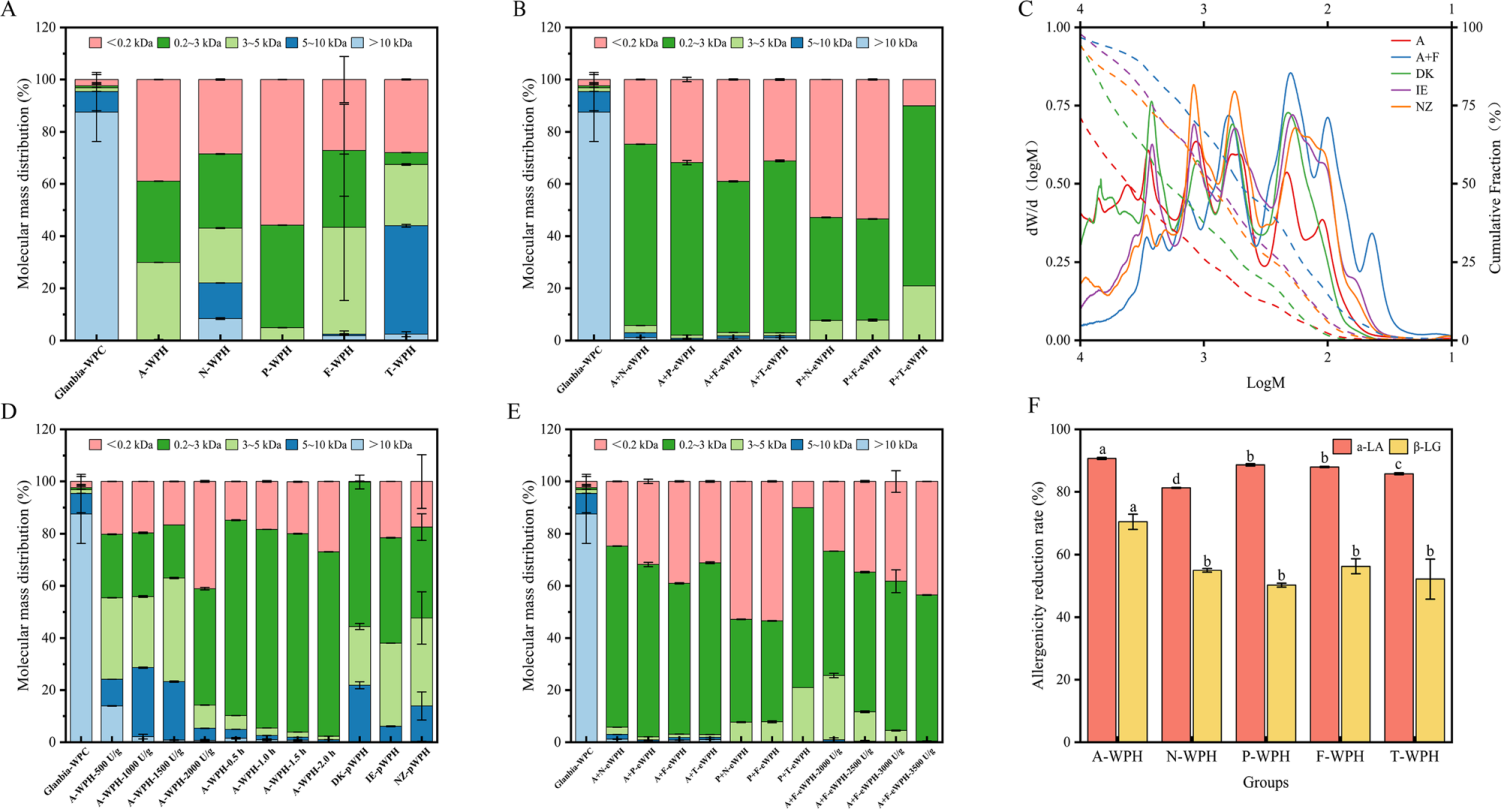
Peptide molecular mass distribution and antigenicity reduction of WPC hydrolyzed by different proteases. **A**, **B**, **D**, **E** Molecular mass distribution of peptides under the hydrolysis conditions of different proteases. **C** Average molecular weight and proportion of products under different hydrolysis conditions and commercially available hydrolyzed peptides. **F** Changes in the antigenicity of peptides under the hydrolysis of different proteases. Data are expressed as mean ± standard deviation (SD, *n* = 3). Different lowercase letters (a, b, c) indicate significant differences among groups (*p* < 0.05, one-way ANOVA with Tukey’s post-hoc test).

The molecular weight distribution results of single-enzyme and combined-enzyme hydrolysis of whey protein concentrate are shown in Fig. 4A, B. It illustrates the hydrolysis efficiency for molecular weight <3000 Da followed the order: papain>alcalase>flavourzyme>neutrase>trypsin. The sandwich ELISA kit used in this study relies on the specific binding of detection antibodies to intact linear epitopes of α-LA and β-LG. For highly hydrolyzed samples, cross-reactivity limitations primarily stem from two key factors: Epitope fragmentation: Excessive hydrolysis cleaves intact allergens into small peptides (<3 kDa), disrupting linear/conformation-dependent epitopes recognized by antibodies. Even when allergen peptide fragments retain residual IgE-binding capacity, this process reduces antibody binding efficiency^26^. Nonspecific binding to minor allergens: Although the assay exhibits low cross-reactivity with minor allergens (lactoferrin, BSA) in intact protein samples, hydrolyzed minor allergen peptides may weakly cross-react with α-LA/β-LG antibodies, potentially leading to a slight overestimation of residual α-LA/β-LG content^19,27^.

Signal suppression is a common phenomenon in ELISA assays for highly hydrolyzed proteins. In this study, its mechanism was primarily attributed to: Peptide competition: Small peptides generated by deep hydrolysis may nonspecifically bind capture/detection antibodies or block antibody binding sites, thereby reducing signal intensity for intact α-LA/β-LG residues^28^. Matrix effects: High concentrations of small peptides and free amino acids in hydrolysates alter the buffer environment (e.g., ionic strength, pH), inhibiting antigen-antibody binding kinetics^29^. Therefore, ELISA cannot fully predict clinical allergies. Fig. 4F, shows the allergenicity reduction results after hydrolysis by the five proteases, among which the alcalase exhibited significant advantages in allergenicity reduction rate, with reductions of 90.73% for α-lactalbumin (α-LA) and 70.48% for β-lactoglobulin (β-LG).

Therefore, based on the results in Fig. 4A, alcalase and Papain were selected to investigate 1:1 compounding with other enzymes. In terms of hydrolysis efficiency for molecular weight <3000 Da, the combinations of alcalase + papain, alcalase + flavourzyme, and alcalase + trypsin showed significant advantages. To ensure a consistent enzyme activity-to-substrate ratio, the amount of each enzyme added varied according to its different enzyme activities. Since papain had relatively low enzyme activity (4000 U/g), a dosage was required during addition, which was not cost-effective. Commercially available trypsin also showed limited advantages, and considering the difficulty in removing bitterness from extensively hydrolyzed whey protein peptides, alcalase was selected for Partial hydrolysis, while a 1:1 combination of alcalase and flavourzyme was chosen for extensive hydrolysis.

According to the molecular weight results of partially hydrolyzed whey protein in Fig. 4D, the optimal partial hydrolysis conditions using alcalase were determined by different hydrolysis times and enzyme-to-substrate ratios. The peptide segments with molecular weight <5 kDa accounted for approximately 80%, with fewer large molecular peptides, achieved under the conditions of an enzyme-to-substrate ratio of 1500 U/g and hydrolysis for 0.5 h. Three partially hydrolyzed whey proteins from Denmark (DK), Ireland (IE), and New Zealand (NZ) were compared with the experimental sample. The molecular weight distribution of these three moderately hydrolyzed whey proteins <5 kDa ranged from 78.16% to 93.89%. Compared with three commercially available partially hydrolyzed whey proteins from DK, IE, and NZ, these conditions met the requirements for partial hydrolysis. Analysis of the results for the average molecular weight and proportion of partially hydrolyzed (A), extensively hydrolyzed (A + F), DK, IE, and NZ peptides (Fig. 4C) reveals that the average molecular weight of A peptides is nearly comparable to those of three commercial hydrolyzed peptides, demonstrating good concordance with the molecular weight characteristics required for functional whey protein peptide-based food ingredients. The average molecular weight of A + F peptides exhibits a distinct and significant scientific advantage, representing a well-characterized, extensively hydrolyzed whey protein fraction with a defined low-molecular-weight profile that is underrepresented in current fundamental research on whey protein hydrolysate structure-function relationships.

Figure 4E presents the molecular weight results of extensively hydrolyzed whey protein, where the proportion of molecules <3000 Da reached approximately 95%, indicating that extensive hydrolysis requirements were satisfied. From the single-enzyme hydrolysis conditions in Fig. 4D, a hydrolysis time of 2 h was found to meet extensive hydrolysis requirements. Hydrolysis significantly reduced allergenicity: α-lactalbumin content decreased by 90.73% and β-lactoglobulin by 70.48% after A-eWPH treatment (Fig. 3F). Furthermore, flavourzyme is compatible with alcalase under mild hydrolysis conditions (pH 7.0–9.5, 50 °C) and offers acceptable industrial costs, making this enzyme combination suitable for large-scale production of hypoallergenic whey protein hydrolysates with an ideal peptide size distribution. As a reference, the dosage of the 1:1 alcalase and flavourzyme combination was optimized. Fig. 4E shows that an enzyme-to-substrate ratio of 3000 U/mg achieved extensive hydrolysis conditions.

The molecular weight is a form that intuitively reflects the effect after enzymatic hydrolysis. This process also further determines the conditions for extensive and partial hydrolysis. Moreover, as can be seen from Table S1 and Table S2, there is no statistically significant loss. The addition of enzymes increases the amount of essential amino acids and the protein similarity, providing parameters for the determination of the calcium content in the subsequent process.

### Calcium transport analysis

**Fig. 5:**
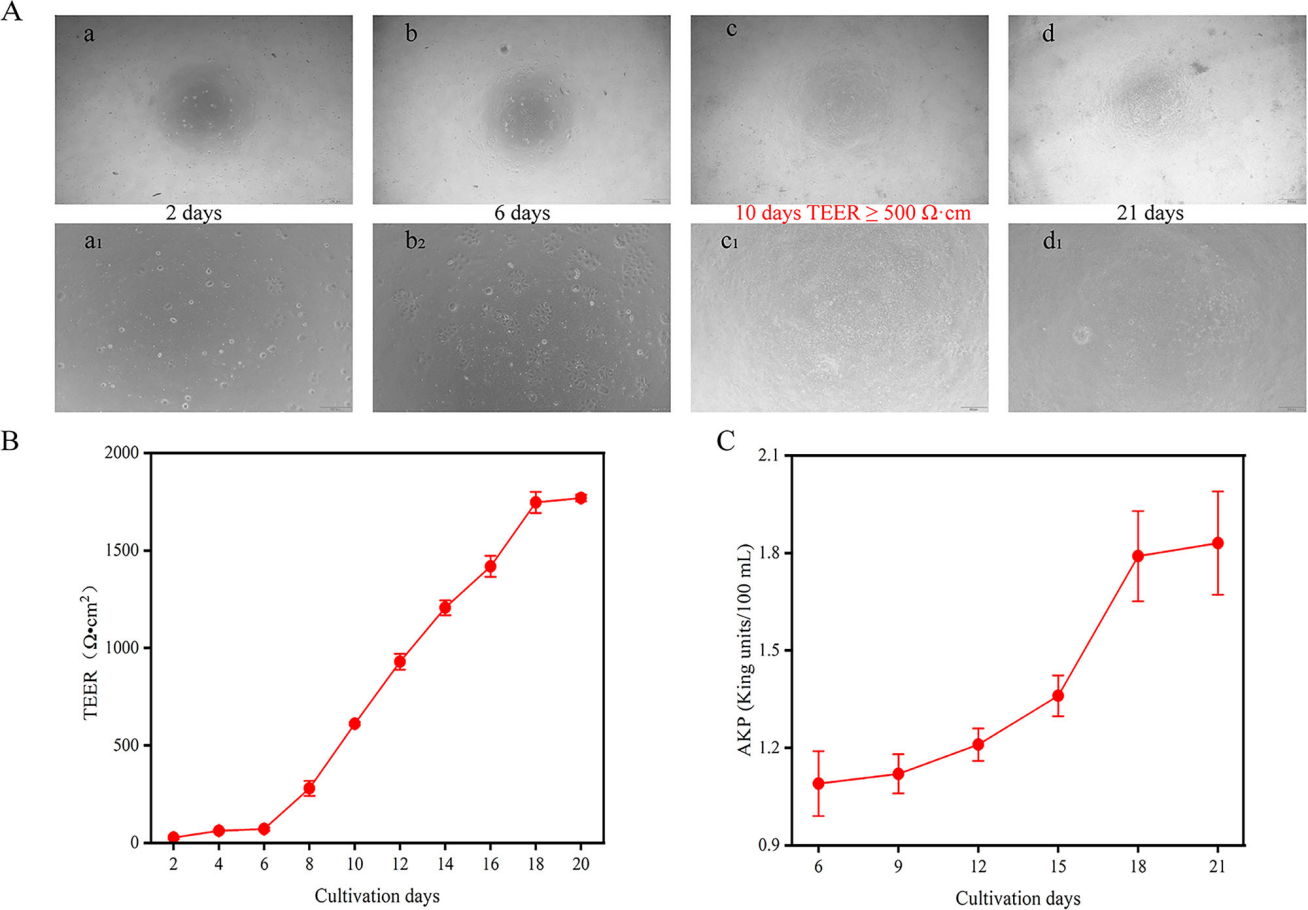
Establishment and characterization of Caco-2 monolayer cell model for in vitro transport studies. **A** Observation diagrams of the morphology of monolayer cells on the 2nd, 6th, 10th, and 21st days (a-d show the cell morphology observed under a 4× microscope, and a1–d1 show the cell morphology observed under a 10× microscope). **B** TEER values of the monolayer cell model on different days. **C** AKP values of the monolayer cell model on different days.

The process of establishing the Caco-2 monolayer cell model for calcium transport analysis is shown in Fig. 5A. After 10 days of culture, the apical membrane was formed. During modeling, transepithelial electrical resistance (TEER) values Fig. 5B and alkaline phosphatase (AKP) activity ratios were monitored Fig. 5C. The maturity of Caco-2 monolayers was assessed by measuring TEER values using an epithelial voltmeter (EVOM2, World Precision Instruments, USA). The acceptance threshold for tight monolayer formation was TEER > 500 Ω cm² (consistent with literature standards^30^). These values steadily increased during the 21-day culture period. As the modeling time approached, no significant decline in TEER was observed, with minimal differences noted between model groups (specific values are provided in Supplementary Table S4). AKP activity in each treatment group was expressed as a ratio relative to the control group, where a ratio >1 indicated enhanced intestinal epithelial cell differentiation. This indicated successful model establishment, enabling subsequent cal cium ion transport experiments.

**Fig. 6:**
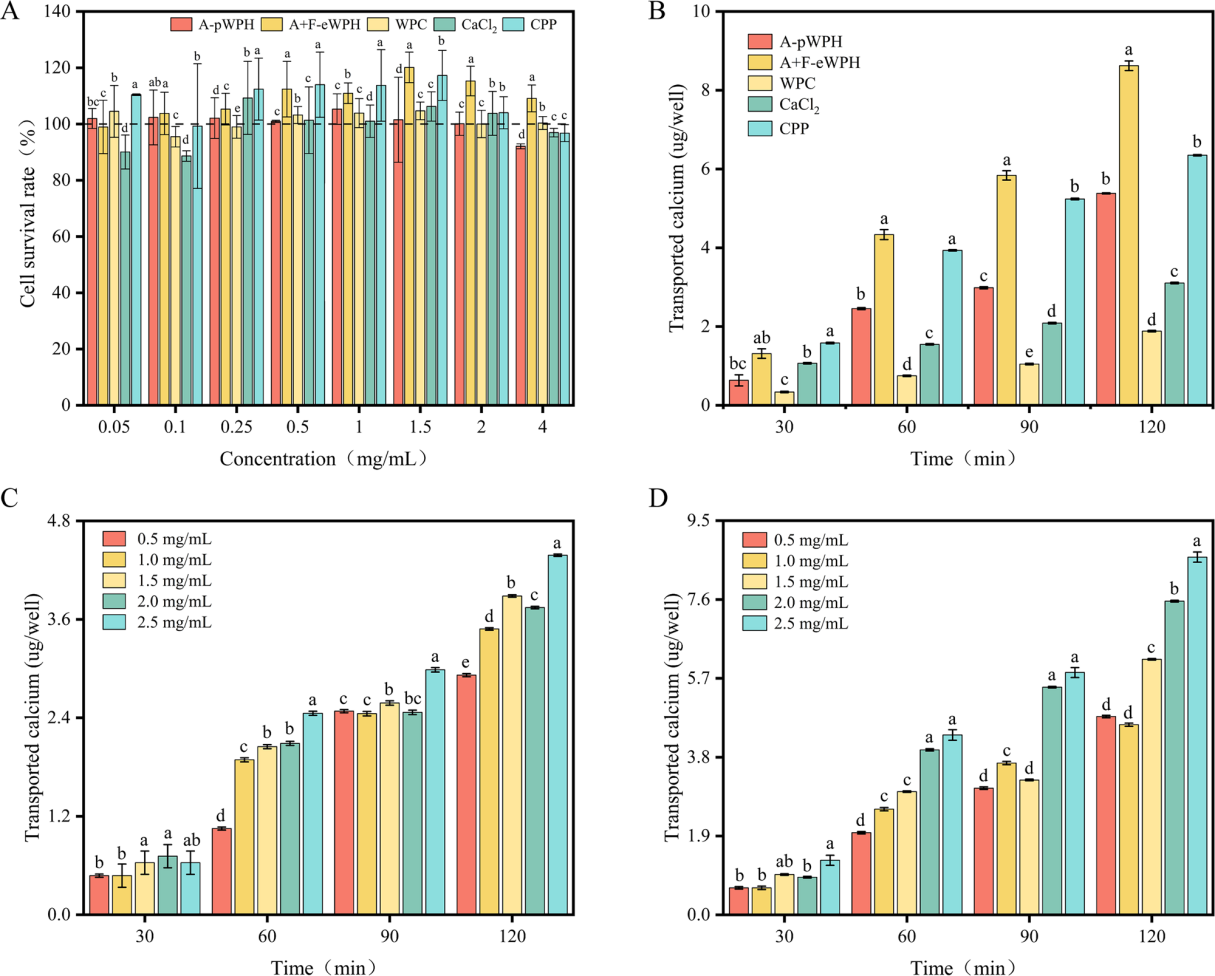
Cytotoxicity and calcium transport activity of hydrolyzed whey protein peptides in Caco-2 monolayer cell model. **A** Cytotoxicity of different samples at different sample concentrations. **B** Calcium transport amounts of A-pWPH, A + F-eWPH, CaCl₂, and CPP-Ca with the same calcium content in the Caco-2 monolayer cell model. **C**, **D** Calcium transport amounts of A-pWPH and A + F-eWPH at different concentrations in the Caco-2 monolayer cell model. Data are expressed as mean ± standard deviation (SD, *n* = 3). Different lowercase letters (a, b, c) indicate significant differences among groups (*p* < 0.05, one-way ANOVA with Tukey’s post-hoc test).

Calcium transport efficiency was normalized based on the Caco-2 monolayer cell area (cm²). Raw transport data (μg/well) are shown in Fig. 6A, while normalized data are presented in Fig. 6B–D. Compared with CPP, A + F-eWPH increased calcium transport efficiency by 35.75% (*p* < 0.05), confirming its superior calcium transport capacity.

Low-molecular-weight peptides (<3 kDa) constitute a high proportion of the peptide chain, exhibiting enhanced hydrophobicity and significantly outperforming CPPs. These small-molecule hydrophobic peptides form stable peptide-calcium chelates via carboxyl (-COOH) and amino (-NH₂) groups, thereby enhancing calcium solubility (reducing calcium precipitation in the intestinal lumen) and promoting Ca^2+^ transport through Caco-2 monolayer cells^30^. They can upregulate mRNA and protein expression of intestinal calcium transport-related channels: TRPV6, PMCA1b, etc., which promote active calcium ion efflux to the basolateral side^31^. It increased the expression levels of tight junction proteins, respectively, compared to the control group (p < 0.05). This enhanced the integrity of the Caco-2 monolayer, reduced paracellular calcium leakage, and moderately increased paracellular calcium transport by regulating tight junction permeability^32,33^.

The toxicity analysis results of samples during calcium transport are shown in Fig. 6A. The effects of different concentrations (0.05–4.0 mg/mL) of A-pWPH, A + F-eWPH, WPC, CaCl₂, and casein phosphopeptide (CPP) on Caco-2 cell viability were evaluated. At lower concentrations, A-pWPH and A + F-eWPH, containing beneficial bioactive peptides, provided nutrients to cells without causing damage and potentially promoted cell growth at appropriate concentrations^34,35^. As sample concentration increased, cell viability decreased. All five samples at 0.5–2.0 mg/mL showed cell viability exceeded 100% relative to the control group, demonstrating non-cytotoxicity and suitability for further calcium transport studies.

Different concentrations of A-pWPH and A + F-eWPH were added to the apical chamber, and calcium ion content in the basolateral chamber was measured after 120 min. As shown in Fig. 5C, with increasing calcium ion concentration, calcium transport into the basolateral chamber increased over time. A-pWPH significantly enhanced calcium transport at concentrations of 1.5, 2.0, and 1.5 mg/mL (*p* < 0.05), with the highest transport observed at 2.5 mg/mL. These results align with previous findings, confirming the successful establishment of a functional Caco-2 monolayer model capable of facilitating drug transport^36,37^.

Subsequently, the calcium transport amounts of A-pWPH, A + F-eWPH, WPC, CaCl₂, and CPP in the Caco-2 monolayer cell model were compared, as shown in Fig. 6B. Under conditions of the same calcium content and concentration, after 120 min of transport, the calcium transport amounts of A-pWPH and A + F-eWPH were significantly higher than that of CaCl₂ (*p* < 0.05). At a concentration of 2.5 mg/mL, the calcium transport amounts of A-pWPH and A + F-eWPH were 1.7-fold and 2.8-fold higher than that of CaCl₂, respectively, indicating that A-pWPH and A + F-eWPH exhibit better calcium transport-promoting abilities compared to CaCl₂. Additionally, differences in calcium transport amounts were compared with CPP^38–40^. As shown in Fig. 5B, compared with CaCl₂, the calcium transport amounts of A-pWPH, A + F-eWPH, and CPP significantly increased at all time points after 30 min (*p* < 0.05). At 120 min, there was no significant difference in calcium transport amount between A-pWPH and CPP (*p* > 0.05), while A + F-eWPH exhibited a significant calcium transport advantage over CPP (p < 0.05). Collectively, it has been demonstrated that A + F-eWPH exhibits a remarkable ability in promoting intestinal calcium transport. Meanwhile, A-pWPH can be comparable to CPP.

## Discussion

The objective was to optimize hydrolysis conditions for low-allergenic, calcium-promoting functional ingredients. By integrating the in vitro calcium absorption promotion, molecular weight changes, and muscle cell proliferation rates of whey protein peptides with different degrees of hydrolysis, the key structural parameters of whey protein peptides were determined. Using the Caco-2 monolayer cell model, the in vitro calcium absorption promotion of partially and extensively hydrolyzed whey protein peptides was found to increase by 1.7-fold and 2.8-fold, while the allergenicity of α-lactalbumin (α-LA) and β-lactoglobulin (β-LG) was significantly reduced by 90.73% and 70.48%. Extensive hydrolysis significantly increased the proportion of small peptides (<3 kDa), reaching 2.6 times that of partially hydrolyzed samples and 30.5 times that of unhydrolyzed whey protein concentrate. Both partial and extensive hydrolysis reduced the allergenicity of major whey allergens α-LA and β-LG, with deep hydrolysis demonstrating greater efficacy. Compared to unhydrolyzed WPC, deep hydrolysis reduced α-LA and β-LG content by 90.73 ± 0.32% and 70.48 ± 2.44%, respectively (*p* < 0.05). In the Caco-2 monolayer cell model, the deeply hydrolyzed product enhanced calcium transport efficiency by 3.6 times compared to unhydrolyzed WPC and exceeded commercially available casein phosphopeptide (CPP) by 1.4 times (*p* < 0.05). This achievement provides concrete technical support for developing hypoallergenic calcium-promoting functional ingredients, offering potential solutions to meet the nutritional needs of individuals at risk of calcium deficiency due to milk protein allergy or lactose intolerance. Subsequent research will focus on in vivo validation of calcium-promoting effects in animal models and the purification of key bioactive peptides to further elucidate their structure-function relationships.

## Methods

### Materials

Whey protein concentrates (WPC 80) were provided by five suppliers: Qingdao Suncare Special Food Co. Ltd. (Qingdao, China), suppliers from the USA, Germany, and New Zealand, respectively. alcalase (138 U/mg), neutrase (54 U/mg), and flavourzyme (17 U/mg) were purchased from Novozymes (Denmark), while papain (4 U/mg) and trypsin (67 U/mg) were obtained from Beijing Solarbio Technology Co., Ltd. (Beijing, China). MEM/EBSS Medium, fetal bovine serum (FBS), non-essential amino acids (NEAA), and 0.25% Trypsin-EDTA (1×) were purchased from Gibco. A penicillin-streptomycin antibiotic cocktail was acquired from Sigma-Aldrich (USA). 30% acrylamide/bis-acrylamide (29:1), Tris-HCl (pH 8.8), ammonium persulfate (APS), bromophenol blue, Coomassie Brilliant Blue R250 staining solution, N, N, N’, N’-tetramethylethylenediamine (TEMED), and gel filtration standards were acquired from Bio-Rad Laboratories, Inc. (Hercules, CA, USA). All chemicals used for electrophoresis were molecular biology grade, and those for high-performance liquid chromatography (HPLC) were HPLC grade. All other reagents were of analytical grade.

### Amino acid content of whey protein

The amino acid composition of the samples was determined using an HPLC-MS/MS system (LC: 1260 Infinity II Prime; MS: AB Sciex API 3200 Q TRAP), employing modifications of established protocols^41,42^ Norleucine served as the internal standard, maintained at a final concentration of 2.5 μmol/L throughout the analysis. Calibration curves for individual amino acids were established across a concentration range of 0.05 to 5 μmol/L, boasting correlation coefficients (*R*²) of 0.995 or higher. The limits of detection (LOD) and quantification (LOQ) were determined to range from 0.01 to 0.03 μmol/L and 0.03 to 0.09 μmol/L, respectively. To evaluate the method’s accuracy, recovery rates were assessed by spiking standard amino acids at low (0.1 μmol/L), medium (1 μmol/L), and high (5 μmol/L) concentrations into the sample matrix, yielding recovery percentages of 92.3% to 105.7%, with relative standard deviations (RSD) below 5%.

Protein precipitant: 10% (w/v) trichloroacetic acid (TCA) solution, prepared by dissolving 10 g TCA in 100 mL ultrapure water. Derivatisation solution: A two-component system consisting of OPA reagent and FMOC reagent. OPA reagent: 0.8 g o-phthalaldehyde (OPA) dissolved in 10 mL ethanol, mixed with 90 mL 0.4 mol/L borate buffer (pH 10.4) and 0.2 mL β-mercaptoethanol. FMOC reagent: 0.1 g 9-fluorenylmethoxycarbonyl chloride (FMOC-Cl) dissolved in 100 mL acetonitrile. Sample pretreatment: 40 mg of whey protein peptide powder (dry weight, protein content ≥78.56%) was accurately weighed and dissolved in 10 mL ultrapure water to prepare a 4 mg/mL sample solution. 1 mL of the sample solution was mixed with 1 mL 10% TCA solution (mass/volume ratio = 1:1), vortexed for 1 min, and centrifuged at 13,200 rpm (4 °C) for 4 min. The supernatant was collected for derivatisation. Derivatisation steps: 10 μL of supernatant/standard solution was mixed with 50 μL OPA reagent, reacted at 55 °C for 2 min, then 20 μL FMOC reagent was added and reacted at 55 °C for another 13 min, with a total derivatisation time of 15 min.

Amino acid nutritional quality was assessed using two complementary methodologies: the fuzzy recognition method^23^ and the ratio coefficient method^43,44^. Egg protein was utilized as a reference protein for the fuzzy recognition method, while the FAO/WHO 2007 reference pattern was employed in the ratio coefficient method. The corresponding key formulas are detailed below^45^.

1 $${\rm{\mu }}\left. (\mathrm{a},{\mathrm{u}}_{\mathrm{i}}\right)=1-0.09\mathop{\sum }\limits_{\mathrm{k}=1}^{7}\frac{\left|{\mathrm{a}}_{\mathrm{k}}-{\mathrm{u}}_{\mathrm{ik}}\right|}{{\mathrm{a}}_{\mathrm{k}}-{\mathrm{u}}_{\mathrm{ik}}}$$

Where *μ* represents the whey protein concentrate to be evaluated, with *μ (μ1, μ2, μ3, μ4, μ5)* corresponding to five types of WPC (Glanbia, HTR, Saputo, Wheyco, Hilmar); ${a}_{k}$ denotes the content of seven essential amino acids (EAA, mg/g pro) in the reference protein (egg protein), where ${a}_{1}$ to ${a}_{7}$ represent leucine (Leu), isoleucine (Ile), lysine (Lys), threonine (Thr), valine (Val), methionine + cysteine (Met+Cys), and phenylalanine + tyrosine (Phe+Tyr), respectively (specific values are listed in Table S1 of the “Results” section); ${u}_{{ik}}$ is the content of the k amino acid in the i whey protein concentrate (mg/g pro).

The amino acid ratio coefficient method calculates the amino acid ratio (RAA), RC, ratio coefficient (SRC), and essential amino acid index (EAAI) based on FAO/WHO reference pattern, using the formulas:

2 $$\mathrm{RAA}=\frac{{\mathrm{E}}_{\mathrm{i}}}{{\mathrm{E}}_{\mathrm{i}0}}$$

3 $${\mathrm{RC}}=\frac{\mathrm{RAA}}{\overline{\mathrm{RAA}}}$$

4 $${\rm{S}}\mathrm{RC}=100\times \left[1-\frac{\sqrt{{\sum }_{\mathrm{i}=1}^{\mathrm{n}}{({\mathrm{RC}}_{\mathrm{i}}-\overline{\mathrm{RC}})}^{2}}}{\overline{\mathrm{RC}}}\right]$$

5 $${\rm{EAAI}}=\root{{7}}\of{\frac{a{a}_{1}\times a{a}_{2}\times a{a}_{3}\times a{a}_{4}\times a{a}_{5}\times a{a}_{6}\times a{a}_{7}}{{\rm{A}}{{\rm{A}}}_{1}\times {\rm{A}}{{\rm{A}}}_{2}\times {\rm{A}}{{\rm{A}}}_{3}\times {\rm{A}}{{\rm{A}}}_{4}\times {\rm{A}}{{\rm{A}}}_{5}\times {\rm{A}}{{\rm{A}}}_{6}\times {\rm{A}}{{\rm{A}}}_{7}}}$$

where ${E}_{i}$ and ${E}_{i0}$ are the contents of the i essential amino acid in the sample and reference pattern (mg/g pro), respectively; $a{a}_{1}$ to $a{a}_{7}$ and $A{A}_{1}$ to $A{A}_{7}$ represent the contents of seven essential amino acids in whey protein concentrate and egg protein (mg/g pro), respectively.

### Optimization of hydrolysis conditions for whey proteins

The optimal hydrolysis conditions for whey protein peptides were determined using a one-way ANOVA. Whey protein samples (5 g) were dissolved in 100 mL of distilled water, and then different proteases (alcalase (138 U/mg), neutrase (54 U/mg), flavourzyme (17 U/mg), papain (4 U/mg), and trypsin (67 U/mg)) were added at an enzyme-substrate ratio of 2000 U/g. The mixtures were incubated at the optimal temperature for each protease, with the pH adjusted to the optimal level using 1 M HCl and NaOH solution. The hydrolysis durations were set at 3 h for the protease screening experiment to ensure complete protein hydrolysis. After the enzymatic hydrolysis, the enzyme was inactivated at 100 °C for 10 min, followed by freezing at −80 °C for over 4 h and vacuum freeze-drying. The dried samples were ground to obtain whey protein peptide powder. Based on the initial screening results and molecular weight data, no statistically significant difference was observed between the 2 h and 3 h samples, for the determination of the enzyme-substrate ratio, whey protein was hydrolyzed by alcalase at pH = 9.5 and 50 °C for 2 h with enzyme-substrate ratios of 500, 1000, 1500, and 2000 U/g. For the determination of the hydrolysis time, under the conditions of pH = 9.5, 50 °C, and an enzyme-substrate ratio of 1500 U/g using alcalase, the hydrolysis time was set to 0.5, 1.0, 1.5, and 2.0 h. The resulting samples were processed in the same manner as above for further analysis.

The normalization principle is based on the target enzyme activity and the specific activity of each protease. The actual mass of enzyme required was calculated using the following formula^46^:

6 $${Enzyme}\,{mass}\left(g\right)=\frac{{Target}\,{enzyme}\,{activity}\left(U/{\rm{g}}\right)\times {Substrate}\,{protein}\,{mass}\left(g\right)}{{Protease}\,{specific}\,{activity}\left(U/{\rm{g}}\right)}$$

Where *Target enzyme activity* = 500, 1000, 1500, and 2000 U/g (relative to substrate protein mass), *Substrate protein mass* = 5 g (WPC sample mass) × 78.56% (Glanbia WPC protein content) = 3.928 g, *Protease specific activity* = the enzymatic activity of each protease.

Based on the experimental results of 2.2.2.1, 2, and 3, a 1:1 mixture of enzymes was selected for the extensive hydrolysis of whey protein. Combinations of alcalase and neutrase, alcalase and papain, alcalase and flavourzyme, alcalase and trypsin, papain and neutrase, papain and flavourzyme, papain and trypsin were chosen. Under the optimal pH and temperature conditions, hydrolysis was carried out at enzyme-to-substrate ratios of 2000 U/g, 2500 U/g, 3000 U/g, and 3500 U/g, with a reaction time of 2 h for each enzyme treatment combination.

### Determination of the hydrolysis degree

The degree of hydrolysis (DH) was measured using the pH-stat method, based on the methods described by refs. ^47,48^, with appropriate modifications. 1 M NaOH solution was used to adjust the pH throughout the hydrolysis process, maintaining the reaction at its optimal pH condition, while continuously recording the volume of NaOH consumed. The specific formula for calculating DH (%) is as follows:

7 $$\mathrm{DH} \% =\frac{\mathrm{B}\times \mathrm{No}}{{\rm{\alpha }}\times {\mathrm{M}}_{\mathrm{p}}\times {\mathrm{h}}_{\mathrm{tot}}}\times 100$$

Where: *B* is volume of NaOH consumed during whey protein peptide hydrolysis (mL); ${No}$ is concentration of NaOH used in whey protein peptide hydrolysis (1 M); $1/\alpha$ is degree of dissociation of α-amino groups which is equal to 2.27^47^; ${Mp}$ is mass of whey protein (g), calculated as sample mass × protein content; ${htot}$ is millimoles of peptide bonds per gram of raw protein (mmol/g), with a value of 8.8 mmol/g for whey protein^47^.

pH was monitored in real time using a pH meter during hydrolysis and adjusted every 15 min. During alcalase hydrolysis (pH = 9.5, prone to fluctuations), the adjustment frequency was increased to every 10 minutes to maintain pH stability within ±0.05 units. 1 M NaOH solution was used, and NaOH consumption was recorded. All hydrolysis experiments (partial and complete hydrolysis) were performed in triplicate (biological replicates, *n* = 3).

### Sodium dodecyl sulfate-polyacrylamide gel electrophoresis (SDS-PAGE)

SDS-PAGE gels were prepared with 12% separating and 5% stacking gels. After adding the separating gel to a glass plate and letting it solidify, the stacking gel was added with a comb inserted. Tetramethylethylenediamine (TEMED) was used for polymerization. Protein sample loading concentration: 2 mg/mL (dissolved in 0.01 M PBS, pH 7.4); loading volume per well: 15 μL. Mix 5×SDS-PAGE loading buffer with protein sample at a 1:4 volume ratio (3 μL loading buffer + 12 μL sample solution), denature by boiling in boiling water at 100 °C for 10 min. Molecular weight standard: Low molecular weight protein marker, load 10 μL per well. Electrophoresis buffer (Tris-glycine buffer, pH 8.3) was added at a constant 300 mL per chamber. Heated in a boiling water bath at 100 °C for 10 min to denature proteins. The prepared gel was placed in an electrophoresis unit, and after adding running buffer, the comb was removed. Five microliters of protein marker (11–245kD) and 20 μL of the sample mixture were loaded into the wells. Electrophoresis was performed at 80 V for 30 min until samples entered the separating gel, after which the voltage was increased to 120 V. Upon completion, the gel was stained with Coomassie rapid staining solution for 30 min, destained until bands were clear, and photographed using an electrophoresis imaging system for analysis.

### Enzyme-linked immunosorbent assay (ELISA)

α-Lactalbumin (α-LA) ELISA Kit (Shandong Meizheng Biotechnology Co., Ltd.): Detection Range = 0.05–5 ng/mL; Limit of Quantification (LOQ) = 0.05 ng/mL; Cross-reactivity rate with other whey proteins (β-LG, lactoferrin) <0.5%. β-Lactoglobulin (β-LG) ELISA Kit (Shanghai Youxuan Biotechnology Co., Ltd.): Detection Range = 0.1–10 ng/mL; Limit of Quantification (LOQ) = 0.1 ng/mL; Cross-reactivity with other whey proteins (α-LA, BSA) < 0.3%. Both employed a double-antibody one-step sandwich ELISA method: pre-coated microplates with β-Lg or α-La antibodies were equilibrated at room temperature (20–25 °C) for ≥30 min. Primary Antibodies: Anti-α-lactalbumin monoclonal antibody (diluted 1:1000, 100 μL/well) and anti-β-lactoglobulin monoclonal antibody (diluted 1:1200, 100 μL/well); Secondary Antibody: HRP-labeled goat anti-rabbit IgG (diluted 1:2000, 100 μL/well). Allergen Standards: α-LA standard (0.05–5 ng/mL) and β-LG standard (0.1–10 ng/mL) were serially diluted using the sample diluent (included in the kit), with 50 μL loaded per well. Add 100 μL/well of substrate solution (TMB) and 50 μL/well of stop solution (2 M H₂SO₄). ELISA working concentration: HRP-labeled substrate (1 U/mL), incubation volume 100 μL/well. All liquid reagents were shaken before use. Set the microplate reader at a wavelength of 450 nm (it is recommended to use a dual-wavelength detection at 450/630 nm, and please read the data within 5 min), and measure the OD value of each well. Take the concentration of the standard substance as the abscissa and the corresponding OD value as the ordinate to draw the linear regression curve of the standard substance. Calculate the concentration values of each sample according to the curve equation. The calculation methods for the decline rates of β-Lg or α-La antigens are the same. Taking β-Lg as an example, the formula is as follows:

8 $$\beta -{Lg}\,{antigenicity}( \% )=\frac{{\mathrm{C}}_{\beta }-{\mathrm{C}}_{\beta -\mathrm{WHP}}}{{\mathrm{C}}_{\beta }}\times 100$$

Where *C*_*β*_ is the concentration of β-Lg in raw whey protein (WPC); *C*_*β-WHP*_ is the concentration of β-Lg in whey protein hydrolysate peptides.

### Size exclusion chromatography (SEC)

The molecular weight (Mw) distribution of whey protein peptides was determined using a Shimadzu high-performance liquid chromatography (HPLC) system equipped with a TSK gel G2000 SWXL column, following a method with appropriate modifications^49^. Evaluating partially and extensively hydrolyzed whey proteins was established based on the obtained Mw distribution. The mobile phase for the experiment consisted of ultrapure water, acetonitrile, and trifluoroacetic acid in a volume ratio of 80:20:0.1. Standard samples and test samples were prepared at a concentration of 1 mg/mL using either the mobile phase or ultrapure water. The standard mixture was composed of cytochrome C (Mw: 12384), aprotinin (Mw: 6511.51), Gly-Gly-Tyr-Arg (Mw: 451.48), L-Gly-Gly-Gly tripeptide (Mw: 189.17), and bacitracin (Mw: 1422.69) in a 1:1:1:1 ratio. Both the standard and test samples were filtered through a 0.22-μm organic membrane into HPLC vials. The detection was carried out using an ultraviolet detector with a mobile-phase flow rate of 0.5 mL/min, a column temperature maintained at 28°C, and an injection volume of 15 μL for 30 min. After injecting the standard sample, a chromatogram was obtained. A standard curve was plotted with the peak-emergence time on the x-axis and the logarithm of the relative molecular weight on the y-axis, from which a regression curve and equation were derived. The retention times for different molecular weights were calculated according to the standard curve. The proportion of the peak area corresponding to each retention time represented the distribution range of the molecular weights.

### Calcium absorption-promoting activity assay

Cell viability was detected using the CCK-8 method^50^. Cells were seeded at a density of 4 × 10^4 to 1 × 10^5 cells/100 μL in 96-well plates and incubated for 24 h. After removing the culture medium, 100 μL of test samples (dissolved in medium) at different WPP concentrations (0.05, 0.1, 0.25, 0.5, 1.0, 1.5, 2.0, 4.0 mg/mL) were added for an additional 24 h incubation. The medium was then aspirated, and 110 μL of CCK-8 mixture (CCK-8: MEM = 1:10) was added to each well. Plates were incubated for 2 h, avoiding bubble formation that could affect OD readings. OD values at 450 nm were measured every 30 min using a microplate reader, with incubation time adjusted according to cell type and experimental needs. Cell viability was calculated as:

9 $$\mathrm{Cell}\,\mathrm{viability}( \% )=\frac{{{\rm{A}}}_{1}-{{\rm{A}}}_{0}}{{{\rm{A}}}_{2}-{{\rm{A}}}_{0}}\times 100$$

where: *A*_1_ is the absorbance of wells with cells, CCK-8 solution, and drug solution; *A*_2_ is the absorbance of wells with medium and CCK-8 solution (no cells); *A*_0_ is the absorbance of wells with cells, CCK-8 solution, and no drug solution.

When cells were in good growth status, they were seeded into 12-well Transwell insert culture plates (pore size 0.4 μm, diameter 12 mm, effective membrane area 1.12 cm²) at a density of 5 × 10^3^ cells/mL. The basolateral (BL) side was filled with 1.5 mL medium, and the apical (AP) side with 0.5 mL medium. Medium was changed every other day for the first week and daily thereafter, with cells cultured for 21–27 days. Cell culture medium color, density, and morphology were observed under an inverted microscope, photographed, and recorded. Transepithelial electrical resistance (TEER) was measured using a Millicell-ERS-2 epithelial volt-ohmmeter^51^. Before measurement, the electrode was sterilized with 75% ethanol–water for 15–30 min and rinsed with a small amount of cell culture medium. TEER was calculated as:

10 $$\mathrm{TEER}=\left({R}_{{\rm{S}}}-{R}_{{\rm{B}}}\right)\times 1.12$$

where 1.12 is the effective membrane area of the 12-well Transwell insert (cm²), *R*_*S*_ is the resistance of the sample well, and *R*_*B*_ is the resistance of the blank control well.

Alkaline phosphatase (AKP) activity was determined using an alkaline phosphatase kit^51^. During medium replacement, 0.5 mL of solution from both AP and BL sides was collected for AKP measurement. The AKP (AP/BL) activity is calculated according to the following formula:

11 $${\rm{AKP}}\left(\frac{{\rm{AP}}}{{\rm{BL}}}\right)=\frac{{{\rm{AKP}}}_{{\rm{AP}}}}{{{\rm{AKP}}}_{{\rm{BL}}}}$$

where AKP_AP_ and AKP_BL_ represent the AKP activity values (King’s units/100 mL) of the solutions on the AP side and the BL side, respectively.

The calcium ion transport was carried out according to the method of ref. ^52^ with some slight modifications. After culturing the Caco-2 monolayer cell model for 21 days, the culture medium was aspirated, and the apical (AP) side was rinsed twice with pre-warmed Hank’s Balanced Salt Solution (HBSS) at 37 °C. Subsequently, the wells were transferred to a new plate containing 1.5 mL of HBSS and incubated in an incubator for 30 min.

After incubation, the HBSS in both the upper and lower wells of the plate was aspirated. Solutions of alcalase partially hydrolyzed whey protein (A-pWPH) and alcalase and flavourzyme extensively hydrolyzed whey protein (A + F-eWPH) at different concentrations (0.5, 1.0, 1.5, 2.0, and 2.5 mg/mL) were respectively added to the AP side, with 0.5 mL for each. Meanwhile, Whey Protein Concentrate (WPC), Calcium Chloride (CaCl_2_), and Casein Phosphopeptide (CPP) were respectively added to the AP side to analyze the effects of different samples with the same calcium content on the calcium transport of Caco-2 cells. Then, 1.5 mL of HBSS was added to the basolateral (BL) side and incubated in the incubator. At different time points (30, 60, 90, and 120 min), 0.5 mL of the solution was aspirated from the BL side for the detection of calcium content, and an equal volume of HBSS was added to maintain a constant volume. The calcium ion content on the BL side measured by the calcium ion kit represents the transported amount.

### Statistical analysis

All experiments were performed in three biological replicates (*n* = 3), with each replicate measured in triplicate (technical replicates). Results are expressed as mean ± SD. Normality was assessed using the Shapiro-Wilk test and homogeneity of variance was tested using Levene’s test. All data met the assumptions of normality (*p* > 0.05) and homogeneity of variance (*p* > 0.05). One-way analysis of variance (ANOVA) and independent samples t-tests were performed on the results. Duncan’s multiple range test was performed using SPSS 25.0 software to determine significant differences, with statistical significance defined as (*p* < 0.05). Different lowercase letters in figures and tables indicate significant differences between data. Graphs were drawn using Origin 2021. All figures were revised to include error bars representing SD (*n* = 3).

## Supplementary information

Supplementary materials

## Data Availability

All data generated or analyzed during this study are included in this published article and its supplementary information files.
