# Supplementary material for: Preparation, allergenicity analysis and nutritional evaluation of partially and extensively hydrolyzed whey protein peptides
Source: NPJ Sci Food. 2026 Mar 10;10:136. doi: 10.1038/s41538-026-00778-8 (PMC13106695; doi:10.1038/s41538-026-00778-8)
Supplement: Supplementary file 1 — Supplementary materials [file 41538_2026_778_MOESM1_ESM.docx]

| **Table S1** Closeness of proteins in five types of WPC and two hydrolyzed peptides to the standard protein. | | | | | | | |
| --- | --- | --- | --- | --- | --- | --- | --- |
| Protein source | Glanbia | HTR | Saputo | Wheyco | Hilmar | A-pWPH | A+F-eWPH |
| To be evaluated | μ1 | μ2 | μ3 | μ4 | μ5 | μ6 | μ7 |
| Closeness degree | 0.801 | 0.773 | 0.800 | 0.787 | 0.794 | 0.880 | 0.880 |

| **Table S2** Comparison of RAA, RC, SRC, and EAAI of essential amino acids in five types of WPC and two hydrolyzed peptides (based on whole-egg pattern). | | | | | | | | | | |
| --- | --- | --- | --- | --- | --- | --- | --- | --- | --- | --- |
| Protein source |  | **Leu** | **Ile** | **Lys** | **Thr** | **Val** | **Met+Cys** | **Phe+Tyr** | **SRC** | **EAAI** |
| mg/g | Egg | 86 | 54 | 70 | 47 | 66 | 57 | 93 |  |  |
| Glanbia | RAA | 0.51 | 0.41 | 1.35 | 1.51 | 0.36 | 0.63 | 0.41 | 39.69 | 0.63 |
|  | RC | 0.69 | 0.55 | 1.82 | 2.04 | 0.49 | 0.85 | 0.55 |  |  |
| HTR | RAA | 0.39 | 0.34 | 1.13 | 1.31 | 0.28 | 0.51 | 0.32 | 35.66 | 0.51 |
|  | RC | 0.64 | 0.55 | 1.84 | 2.14 | 0.46 | 0.84 | 0.52 |  |  |
| Wheyco | RAA | 0.45 | 0.38 | 1.22 | 0.95 | 0.32 | 0.52 | 0.38 | 47.47 | 0.54 |
|  | RC | 0.75 | 0.63 | 2.01 | 1.58 | 0.54 | 0.86 | 0.63 |  |  |
| Hilmar | RAA | 0.46 | 0.39 | 1.37 | 1.11 | 0.33 | 0.43 | 0.38 | 38.99 | 0.55 |
|  | RC | 0.72 | 0.62 | 2.15 | 1.74 | 0.51 | 0.67 | 0.60 |  |  |
| Saputo | RAA | 0.41 | 0.36 | 1.19 | 1.41 | 0.30 | 0.76 | 0.35 | 39.14 | 0.57 |
|  | RC | 0.60 | 0.53 | 1.74 | 2.05 | 0.44 | 1.12 | 0.52 |  |  |
| A-pWPH | RAA | 0.76 | 0.81 | 1.02 | 1.10 | 0.56 | 0.48 | 0.48 | 68.52 | 0.71 |
|  | RC | 1.02 | 1.09 | 1.37 | 1.48 | 0.76 | 0.64 | 0.64 |  |  |
| A+F-eWPH | RAA | 0.75 | 0.80 | 0.82 | 1.08 | 0.54 | 0.46 | 0.51 | 71.53 | 0.68 |
|  | RC | 1.06 | 1.13 | 1.15 | 1.52 | 0.76 | 0.65 | 0.73 |  |  |

| **Table S3** Comparison of RAA, RC, SRC, and EAAI of essential amino acids in five types of WPC and two hydrolyzed peptides (based on FAO/WHO pattern). | | | | | | | | | | |
| --- | --- | --- | --- | --- | --- | --- | --- | --- | --- | --- |
| Protein source |  | **Leu** | **Ile** | **Lys** | **Thr** | **Val** | **Met+Cys** | **Phe+Tyr** | **SRC** | **EAAI** |
| mg/g | FAO/WHO | 70 | 40 | 55 | 40 | 50 | 35 | 60 |  |  |
| Glanbia | RAA | 0.62 | 0.55 | 1.71 | 1.78 | 0.48 | 1.03 | 0.63 | 47.01 | 0.85 |
|  | RC | 0.64 | 0.57 | 1.76 | 1.83 | 0.49 | 1.06 | 0.65 |  |  |
| HTR | RAA | 0.48 | 0.45 | 1.44 | 1.54 | 0.38 | 0.84 | 0.49 | 43.23 | 0.69 |
|  | RC | 0.60 | 0.57 | 1.79 | 1.92 | 0.47 | 1.04 | 0.62 |  |  |
| Wheyco | RAA | 0.56 | 0.51 | 1.55 | 1.12 | 0.43 | 0.84 | 0.59 | 53.09 | 0.73 |
|  | RC | 0.69 | 0.64 | 1.93 | 1.40 | 0.54 | 1.06 | 0.74 |  |  |
| Hilmar | RAA | 0.56 | 0.53 | 1.75 | 1.30 | 0.43 | 0.69 | 0.59 | 45.46 | 0.74 |
|  | RC | 0.67 | 0.63 | 2.09 | 1.56 | 0.51 | 0.83 | 0.71 |  |  |
| Saputo | RAA | 0.51 | 0.49 | 1.52 | 1.65 | 0.40 | 1.25 | 0.55 | 44.79 | 0.78 |
|  | RC | 0.56 | 0.54 | 1.67 | 1.82 | 0.44 | 1.37 | 0.60 |  |  |
| A-pWPH | RAA | 0.93 | 1.10 | 1.29 | 1.29 | 0.74 | 0.77 | 0.93 | 76.58 | 0.96 |
|  | RC | 0.95 | 1.12 | 1.32 | 1.32 | 0.76 | 0.79 | 0.75 |  |  |
| A+F-eWPH | RAA | 0.92 | 1.09 | 1.04 | 1.27 | 0.72 | 0.75 | 0.92 | 80.71 | 0.92 |
|  | RC | 0.98 | 1.16 | 1.11 | 1.35 | 0.76 | 0.80 | 0.85 |  |  |

| **Table S4** Resistivity Values During the Cell Cycle in the Caco-2 Cell Model | |
| --- | --- |
| days | TEER(Ω·cm²) |
| 2 | 26.67±7.64 |
| 4 | 61.67±7.64 |
| 6 | 70.67±8.50 |
| 8 | 279.33±38.85 |
| 10 | 611.00±7.81 |
| 12 | 929.00±40.58 |
| 14 | 1206.00±37.75 |
| 16 | 1418.33±53.78 |
| 18 | 1746.33±54.50 |
| 20 | 1768.67±16.92 |

Scanned copies of molecular weight/size markers shown in Fig.S2C and Fig.S2D


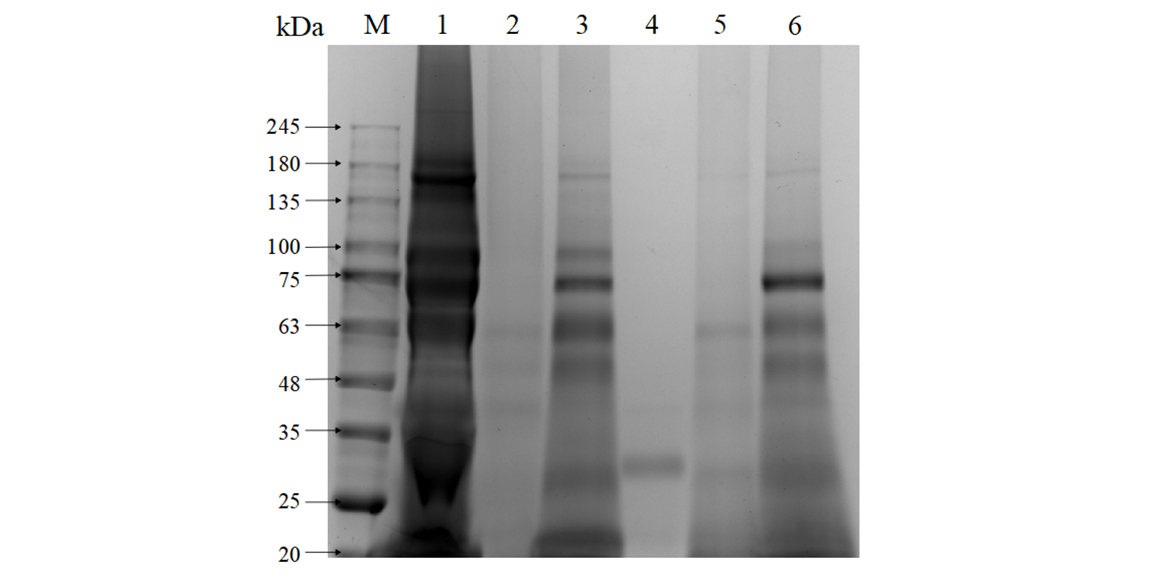

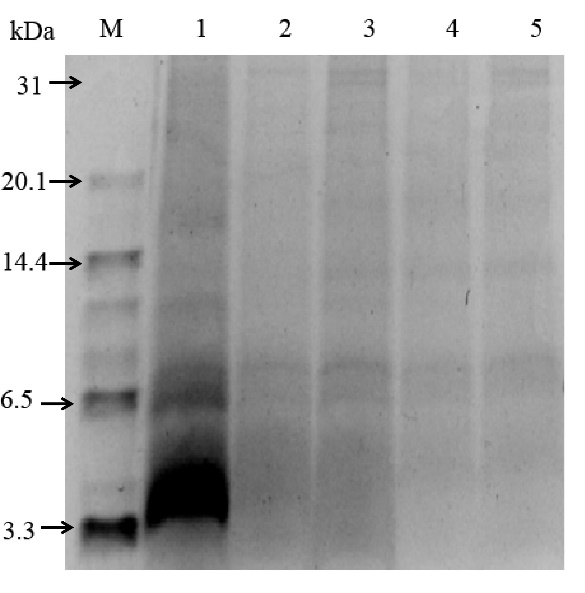


Uncropped and unprocessed scans of Fig.S2C and Fig.S2D

**
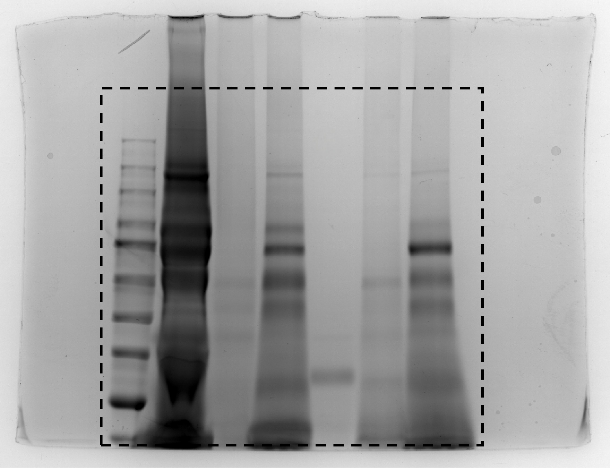

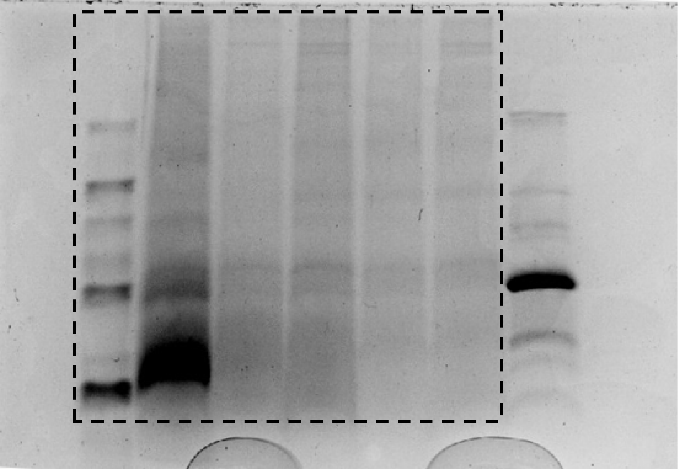
**
